# Rapid and sensitive identification of omicron by variant-specific PCR and nanopore sequencing: paradigm for diagnostics of emerging SARS-CoV-2 variants

**DOI:** 10.1007/s00430-022-00728-7

**Published:** 2022-01-21

**Authors:** Christopher Dächert, Maximilian Muenchhoff, Alexander Graf, Hanna Autenrieth, Sabine Bender, Helga Mairhofer, Paul R. Wratil, Susanne Thieme, Stefan Krebs, Natascha Grzimek-Koschewa, Helmut Blum, Oliver T. Keppler

**Affiliations:** 1 Max von Pettenkofer Institute & Gene Center, Virology, National Reference Center for Retroviruses, LMU München, Pettenkoferstr. 9a, 80336 Munich, Germany; 2 German Center for Infection Research (DZIF), Partner Site Munich, Munich, Germany; 3 Laboratory for Functional Genome Analysis, Gene Center, LMU München, Munich, Germany

**Keywords:** SARS-CoV-2, VOC, VOC-PCR, Nanopore, Omicron, Melting curve analysis

## Abstract

On November 26, 2021, the World Health Organization classified B.1.1.529 as a severe acute respiratory syndrome coronavirus 2 (SARS-CoV-2) variant of concern (VoC), named omicron. *Spike*-gene dropouts in conventional SARS-CoV-2 PCR systems have been reported over the last weeks as indirect diagnostic evidence for the identification of omicron. Here, we report the combination of PCRs specific for heavily mutated sites in the *spike* gene and nanopore-based full-length genome sequencing for the rapid and sensitive identification of the first four COVID-19 patients diagnosed in Germany to be infected with omicron on November 28, 2021. This study will assist the unambiguous laboratory-based diagnosis and global surveillance for this highly contagious VoC with an unprecedented degree of humoral immune escape. Moreover, we propose that specialized diagnostic laboratories should continuously update their assays for variant-specific PCRs in the *spike* gene of SARS-CoV-2 to readily detect and diagnose emerging variants of interest and VoCs. The combination with established nanopore sequencing procedures allows both the rapid confirmation by whole genome sequencing as well as the sensitive identification of newly emerging variants of this pandemic β-coronavirus in years to come.

## Introduction

Already in the last weeks of December 2021, omicron has become the dominant variant of concern (VoC) in many countries, including Great Britain, France, Denmark, Greece, Spain, Portugal, and the US [1] with numbers of new infections reaching all-time highs. Earlier VoCs were characterized either by an increased ability for transmission in the population [VoCs alpha (B.1.1.7) and delta (B.1.617.2)] or a partial immune escape with variable effects on neutralization by polyclonal serum antibodies [VoCs beta (B.1.351), gamma (P.1/B.1.1.28) and delta] [2–7]. A striking characteristic of the apparently independently evolved omicron VoC is a large number of amino acid substitutions, insertions and deletions in the spike protein, i.e. 32 changes compared to the original Wuhan-hu-1 virus [8], that likely contribute to its extraordinarily rapid spread in the population. Moreover, the number of epitopes in the spike protein relevant for neutralization are an important determinant of the genetic barrier to viral escape from humoral immunity [6, 9]. Consequently, physician-scientists have been alerted since its discovery based on the genetic information alone by omicron’s potential for a pronounced immune escape. Recent laboratory studies and epidemiological reports have now confirmed this exceptional combination of high contagiousity and drastic escape from neutralizing antibodies [10–12]. The resistance to neutralization is most likely due to mutations K417N, N440K, G446S, S477N, T478K, E484A, Q493R, G496S, Q498R, N501Y and Y505H, which are located within or close to the epitopes bound by these antibodies [13].

Here, we report the laboratory-based diagnosis of the first four COVID-19 cases with an omicron infection reported in Germany based on a combination of variant-specific PCRs and next-generation-sequencing using an established nanopore platform.

## Materials and methods

### Patients and collection of respiratory samples for testing for SARS-CoV-2

Respiratory samples (either oropharyngeal gargle or nasopharyngeal swab) were collected in Bio-Speedy vNAT Transfer Tubes (Bioeksen R&D Technologies, Cat. No. BS-NA-513-100) from patients on November 27, 2021, who had self-reported a positive SARS-CoV-2 PCR result two days after returning from Cape Town, South Africa, following a two-week round trip (patient 1 and 2), or patients arriving in Germany at Munich airport on a flight from Cape Town, South Africa, on November 26, 2021 (patient 3 and 4). For additional patient characteristics and sample details see Table 1.

**Table 1:** Patient characteristics, sample details and results

Patient ID	Age	Sex	Swab (s)/oropharyngeal gargle (g)	Ct	Viral load (Geq/ml)	VOC-PCR (11/27/2021)	Nanopore sequencing (11/28/2021)	GISAID Accession ID	Mean coverage	*N* percentage
1	66	m	11/27/2021 (g)	27.2	160,000	B.1.1.529	B.1.1.529	EPI_ISL_6886594	417	1.33
2	64	f	11/27/2021 (g)	29.1	33,000	B.1.1.529	B.1.1.529	EPI_ISL_6886593	425	0.96
3	34	m	11/26/2021 (s)	23.2	n.d	B.1.1.529	B.1.1.529	EPI_ISL_6886595	553	1.05
4	28	m	11/26/2021 (s)	31.3/neg	n.d	indeterminate	B.1.1.529	EPI_ISL_6886596	94	4.96

Clinical characteristics of patients (age in years and sex) are indicated. Collection dates and cycle threshold (Ct) values are shown for the nasopharyngeal swabs and oropharyngeal gargle samples. Viral load is expressed as SARS-CoV-2 *nucleocapsid* copy numbers per milliliter of oropharyngeal gargle. Pangolin lineage classification is indicated as based on VOC-PCR or nanopore sequencing analysis performed on November 27 and 28, 2021, respectively. Accession numbers are indicated for sequences deposited at the global initiative on sharing avian flu data (GISAID). The average reads per nucleotide (mean sequence coverage) and ambiguity (percentage of nucleotide positions that are unresolved, *N* percentage) are shown

### Quantitative viral load determination

Samples were quantified for SARS-CoV-2 RNA using the fully-automated Roche cobas^®^ 6800/8800 system (Roche, Mannheim, Germany). For quantification, standard curves were generated in multiple replicates using a commercially available standard for calibration (Instand e.V.) as described previously [14]. Viral loads of samples were calculated as SARS-CoV-2 *nucleocapsid* gene copy numbers per 1 ml of oropharyngeal gargle.

### SARS-CoV-2 VOC-specific PCRs

**Fig. 1:**
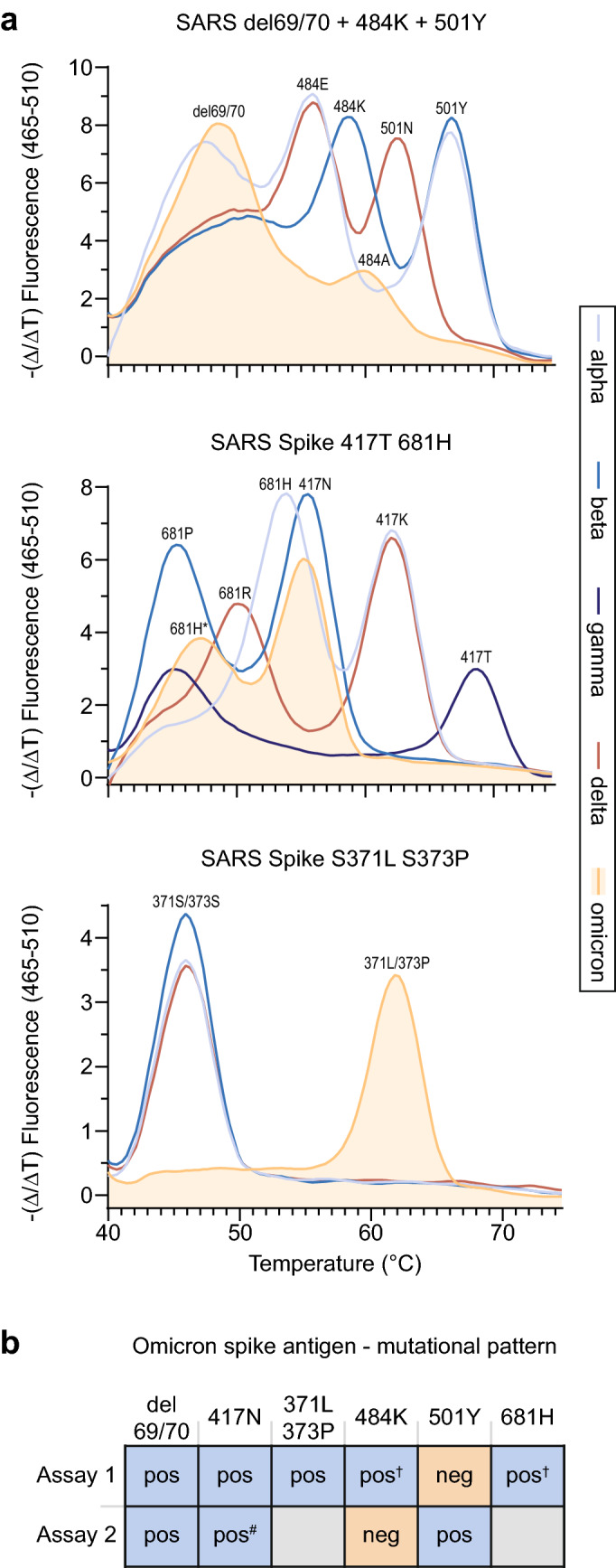
Detection of omicron (B.1.1.529) by SARS-CoV-2 variant-specific PCRs for the *spike* gene. **a** PCR probe-based melting curve assays (ref 26) for the identification of SARS-CoV-2 VOCs. Melting curves for spike mutations/deletions at the indicated amino acid positions. Data for all five VOCs are depicted. **b** Summary of mutational pattern for omicron detected by two different VOC-specific assays (Assay 1: TIB MOLBIOL (ref 26); Assay 2: Seegene Variants I/II (refs 27, 28). ^†^ Melting curve peak shifted and dampened. ^#^Delayed Ct value

Nucleic acids from patients’ respiratory samples were extracted using the QIAsymphony DSP Virus/Pathogen Kit (cat. no. 937036) on a QIAsymphony SP instrument or the EZ1 Virus Mini Kit v2.0 (cat. no. 955134) with the EZ1 Advanced XL (Qiagen, Hilden, Germany). Eluates were used for melting curve analysis performed on a LightCycler480 II (Software version 1.5.1.62) (Roche Diagnostics, Basel, Switzerland) with the Luna^®^ Probe One-Step RT-qPCR Kit (No ROX) (cat. no. E3007E, NEB, Frankfurt am Main, Germany) and the following VirSNiP kits (TIB MOLBIOL, Berlin, Germany): SARS del69/70 + 484 K + 501Y (Cat. No. 53-0799-96) (Fig. 1A, top panel), SARS Spike 417 T 681H (Cat. No. 53-0807-96) (Fig. 1A, middle panel) and SARS Spike S371L S373P (53-0827-96) (Fig. 1a*,* lower panel). We followed the manufacturer instructions for the VirSNiP assay except adapting the thermocycler program to the Luna^®^ Probe One-Step RT-qPCR mix with the following modification: we shortened the 60 °C step to 15 s and added a step at 72 °C for 15 s in every cycle. Seegene Variants I and II assays (cat. no. RV10286X and RV10305X) were performed on C1000 Thermal Cyclers (Bio-Rad, Feldkirchen, Germany) according to the manufacturer’s instructions.

### SARS-CoV-2 whole-genome sequencing

Sequencing libraries were prepared according to the ARTIC network nCoV-2019 sequencing protocol v3 [15, 16]. cDNA was generated from extracted total RNA using LunaScript RT (NEB, Ipswich, USA) and amplified with ARTIC primer set v3 [17]. The v3 primer set was rebalanced to achieve a more uniform amplicon coverage [18] and supplemented with updated versions of primer 64_left (ATGTCGATAGATATCCTGTTAATTCCATTGT) and 72_right (CTAGAATAAACTCCACTTTCCATCCAAC) to avoid lineage-specific dropouts. The library quality was routinely checked in each run using standardised control samples. PCR amplification was as described [15, 16] with 35 cycles and 64 °C annealing temperature. PCR reactions were diluted 1:10 and the two primer pool reactions for each sample were pooled. Diluted amplicons were end-repaired, purified with Ampure XP beads, ligated to barcode adapters (Oxford Nanopore Technologies (ONT), Oxford, UK), purified again with Ampure XP beads, pooled and ligated to AMII sequencing adapter (ONT, Oxford, UK). The purified library was sequenced on a PromethION R9.4.1 flowcell until sufficient coverage for lineage calling was reached, monitored by RAMPART software package [19].

Sequencing reads were basecalled with the Guppy basecaller (v.5.0.17) using the super-accurate DNA model provided by ONT (Oxford, UK). The samples were demultiplexed according to the EXP-NBD196 barcode kit. The sequenced amplicons were assembled using the ARTIC bioinformatics protocol [20]. Following the protocol, the artic tool was used to filter the reads by the quality and by a read length between 380 and 700 nucleotides. Reads were mapped to the Sars-Cov-2 reference genome (NC_045512.2) to call variants. Called variants were integrated into the reference sequence to obtain the consensus sequence of the sequenced sample. The lineage assignment was performed with the pangolin tool (v.3.1.16) [21].

## Results and discussion

In four individuals returning from South Africa on November 24 or 26, 2021 to Munich, Germany, SARS-CoV-2 was detected in respiratory swabs by PCR on November 27, 2021 with cycle-threshold (ct) values ranging from 23.2 to 31.2 (Table 1). *Spike*-gene dropouts (or *spike*-gene target failure, SGTF) in conventional PCR systems, which can present either as a markedly delayed Ct value for or an entirely absent detection of the SARS-CoV-2 *spike* gene, have been reported for B.1.1.529 over the last weeks [22–24] as indirect diagnostic evidence for its identification. Typically, non-*spike* gene-targeted sequences, e.g. in the *nucleocapsid* gene, *envelope* gene or *RNA-dependent RNA polymerase gene* [25], performed in these omicron-containing samples in parallel, can be readily amplified.

High-resolution probe-based melting curve assays [26] and multiplex-based PCR assays [27, 28] have allowed simultaneous detection of discriminatory mutation sites for identification and differentiation of VOCs alpha [B.1.1.7], beta [B.1.351], gamma [P.1], and delta [B.1.617.2 and AY]. These tests’ applicability to the identification of omicron [B.1.1.529] had not been reported at the time of this study in late November 2021.

Based on the patients’ recent travel history to South Africa and available sequences for B.1.1.529 from GISAID [29], we employed SARS-CoV-2 variant-specific PCR systems (Assays 1 and 2) from two globally operating commercial vendors (TIB MOLBIOL, Berlin, Germany, and Seegene, Germany, Düsseldorf, Germany) [26–28], which had at the time either not been officially launched or only recently  been launched, on November 27, 2021: RT-PCR products of samples from patients 1–3 (Table 1) showed a peak for del 69/70 (like alpha; unlike beta, gamma and delta) and a shifted and dampened peak for 484 compared to the other VOCs (Fig. 1a (top panel), Tables 2, and 3). The peak for 501 was lost, likely due to surrounding mutations (G496S, Q498R, Y505H). A peak was detected for 417N (like beta; unlike alpha, gamma and delta), and a shifted and dampened peak for 681H (Fig. 1a (middle panel), Tables 2, and 3) (unlike all other VOCs), the latter likely due to omicron’s N679K mutation. Also, a distinct melting curve for 371L/373P compared to alpha, beta and delta was observed (Fig. 1a (lower panel), Tables 2, and 3) [26]. Results for the sample from patient 4 were indeterminate in both assays (Fig. 1b), likely due to the very low virus load in this respiratory sample (ct value of 31.3 or negative; Table 1). Figure 1b summarizes the results for the VOC-specific assays for omicron (see Table 3 for primary output data for Assay 2 for all VOCs [27, 28]).

**Table 2:** Summary of amino acid mutations in spike relevant for VOC identification

	69/70	417	452	484	501	681	371/373
Omicron	del	N	L	A	Y*	H^#^	L/P
Alpha	del	K	L	E	Y	H	S/S
Beta	H/V	N	L	K	Y	P	S/S
Gamma	H/V	T	L	K	Y	P	S/S
Delta	H/V	K	R	E	N	R	S/S

Amino acid positions in the SARS-CoV-2 spike protein critical for the identification of VOCs alpha, beta, gamma, delta and omicron are depicted

*Melting curve peak not detected in assay A

^#^Peak shifted due to surrounding mutations

**Table 3:** Summary of diagnostic findings for spike mutations relevant for VOC identification

	del69/70	417N	452R	484K	501Y	681H	S371L/S373P
Omicron	✓	✓	×	✓*	×	✓*	✓
Alpha	✓	×	×	×	✓	✓	×
Beta	×	✓	×	✓	✓	×	×
Gamma	×	×	×	✓	✓	×	×
Delta	×	×	✓	×	×	×	×

*Peak shifted and/or dampened due to surrounding mutations

Next, RNA extracts of patients’ samples were studied by Nanopore sequencing using the ARTIC protocol version 3 [15, 16], which contains 98 primer pairs designed to cover the entire SARS-CoV-2 genome with overlapping 400-bp amplicons. In less than 12 h, sequencing results and SARS-CoV-2 Pangolin lineage calling were completed (GISAID accession ID in Table 1). Some mutations in the *spike* gene of the omicron VOC result in mismatches for primers used in amplicon generation. For example, amplicon number 76 of the Artic protocol v3/v4 is affected by *spike* mutations A23040G, G23048A and A23055G, which results in reduced coverage of the *spike* region including the amino acid mutations K417N, N440K and G446S. Modified protocols that include additional primers matching these polymorphic sites have been developed and will be implemented in future versions [30]. Of particular note, also the sample of patient 4 with very low/negative virus load as assessed by PCR could now be identified as B.1.1.529 with a genome coverage of 95.04%. This indicates that nanopore sequencing may allow a rapid, highly sensitive determination of the SARS-CoV-2 lineage, including omicron. Compared to established short-read sequencing technologies such as Illumina, the long-read nanopore technology allows more cost-effective processing of smaller batches of samples and offers shorter turnaround times. However, the analytical accuracy of nanopore sequencing is limited for the detection of short indels and variants at low read-count frequencies [31]. For larger batches of samples, where turnaround time is not critical, other sequencing technologies could be more cost-effective.

In conclusion, we report the combination of PCRs specific for heavily mutated sites in the *spike* gene and nanopore-based full-length genome sequencing for the rapid and sensitive identification of the first four COVID-19 patients diagnosed in Germany to be infected with omicron. These findings will assist unambiguous laboratory-based clinical diagnosis and global surveillance for this highly contagious VOC, which carries spike mutations conferring reduced COVID-19 vaccine efficacy. Moreover, specialized diagnostic laboratories should continuously update their assays for variant-specific PCRs in the *spike* gene of SARS-CoV-2 to readily detect and diagnose emerging variants of interest and VoCs. The combination with established nanopore sequencing procedures allows both the rapid confirmation by whole genome sequencing as well as the sensitive identification of newly emerging variants of this pandemic β-coronavirus in years to come.

## Data Availability

Not applicable.
